# Three-Dimensional Printing for Precision and Personalized Patient Care: A New Paradigm for Pharmacy Practice?

**DOI:** 10.3390/pharmaceutics18020158

**Published:** 2026-01-26

**Authors:** Preshita Desai, Katherine Bang, Jeffrey Wang, Patrick Chan, Donald Hsu, Micah Hata, Sunil Prabhu

**Affiliations:** College of Pharmacy, Western University of Health Sciences, Pomona, CA 91766, USA

**Keywords:** 3D printing, super-compounding, polypill, precision medicine, personalized medicine

## Abstract

**Objectives:** Personalized medicine is gaining rapid attention over the current drug prescription approach of ‘one-size-fits-all’. Three-dimensional (3D) printing is one such product development technique that has the potential to transform the pharmaceutical and biomedical sectors. **Methods:** To establish the future of 3D printing in mainstream pharmacy practice, initially, pharmaceutical preclinical and clinical scientific databases (peer-reviewed articles, patents, and marketed products) over the past 10 years were critically scrutinized. Additionally, to provide context, we developed a hypothetical case study illustrating the capabilities of the 3D printing super-compounding pharmacy in personalized patient care, emphasizing the critical role of pharmacists in this process. **Results:** Acknowledging the potential of 3D printing in pharmacy practice, this review effectively summarizes the advances and opportunities of pharmaceutically feasible 3D printing methods, as well as the challenges in translating this technology into a future super-compounding pharmacy facility. Furthermore, the review highlights the promising capabilities of such pharmaceutical 3D printers in enabling on-site printing of 3D medicines tailored to individual needs, which may range from dose adjustments to multidrug single tablets (polypills). **Conclusions:** We believe that 3D printing technology has the potential to revolutionize precision and personalized medication approaches in pharmacy practice, which will significantly benefit patient healthcare outcomes. Additionally, the adoption of such technology in pharmacies will lead to a reinvention of the role of pharmacists, thereby creating more job opportunities. Ultimately, 3D printing will create a new paradigm of super-compounding pharmacy practice, providing a new sense of excitement for those looking to enter the pharmacy profession.

## 1. Introduction

Personalized medicine and patient-focused precision pharmaceutical product development have been gaining rapid attention in recent times. These remarkable shifts are attributed to key advances in personalized medicine, supported by clinical evidence for the pharmacogenomics and epigenomics bases of various diseases. These studies demonstrate that not only disease susceptibility but also drug response may differ from individual to individual due to slight genomic variations. Furthermore, other physiological and pathological parameters like sex, age, weight, prior medical history, side effects, etc., may require an alteration in drug doses from those of the commercially available standard dose treatments. For example, pediatric patients may require only a small dose compared to adults [1]. Similarly, an adult patient weighing 150 lbs may only need a fraction of a dose compared to another adult patient weighing 250 lbs; yet, in the current clinical practice scenario, the prescription may list the same dose for both adults. These issues have underscored the need for personalized medicine, which enables the tailoring of drugs and their doses based on individual parameters. However, the current pharmaceutical industry is primarily based on mass drug product manufacturing, which runs on the principle of ‘one-size-fits-all,’ thereby limiting the clinical translation of personalized medicine. Hence, with an increase in awareness and demand for personalized medicine, it would be very timely to develop custom-made products for precise and personalized use.

Three-dimensional (3D) printing is one such product development technique that is transforming the pharmaceutical and biomedical sectors with a wide range of applications that include but are not limited to drug products, biomedical devices, prostheses, implants, tissue bioengineering, and disease modelling, etc.

Currently, most 3D printed products (over 100) approved by the U.S. Food and Drug Administration (USFDA) are medical devices. Notably, the first 3D printed pharmaceutical drug product, SPRITAM^®^ (levetiracetam), received USFDA approval in 2015. Since then, a handful of 3D printed drug products have entered clinical development. Among these, Triastek Inc. has received USFDA Investigational New Drug (IND) approval and has advanced into clinical trials for their melt extrusion deposition (MED)-based 3D printed drug products, T20G (gastroretentive drug product), and T21 (colon-targeted drug product), demonstrating the potential of 3D printing for controlled and site-specific oral drug delivery. Furthermore, FabRx Ltd. has developed semisolid extrusion-based 3D-printed capsules and other unit dosage forms. Their early community pharmacy trials have shown promising results, and these products are anticipated to advance in clinical trials in the near future. To summarize, these advancements highlight the significant potential and future scope of 3D printing technologies in pharmaceutical drug product development. This is also supported by market projections, which estimate that the pharmaceutical 3D printing market will grow at a cumulative annual growth rate (CAGR) of 6.5% from 2020 to 2030, reaching an estimated market value of $522 million by 2030 [2,3,4,5].

Technically, 3D printing is broadly understood as an additive manufacturing technique that involves layer-by-layer printing of an object. More specifically, the International Standard Organization (ISO) defines the term 3D printing as ‘fabrication of objects through the deposition of a material using a print head, nozzle, or another printer technology’. Among various pharmaceutical applications of 3D printing, drug delivery formulation products can be visualized as a branch that deals with the development of safe and effective medicines for both mass production and personalized prescriptions, making it essential for both industry and pharmacy practice professionals. Specifically, pharmaceutical 3D printers have the capability of finding an ideal place in pharmacy medication therapy management, especially in the ambulatory care and precision compounding operations. This technology will enable on-site printing of medicines tailored to individual patient needs, ranging from dose adjustments for specific patients to multidrug single tablets (polypills). Therefore, for pharmacy practice professionals, 3D printing offers a unique and novel strategy that enables personalized medicine development tailored to patient-specific needs [6,7].

In view of this, we believe that it is more important, now more than ever, to thoroughly understand 3D printing technology, its applications in pharmaceuticals, and, most importantly, to understand the technology from a pharmacy practice viewpoint, which is the scope of this review. Specifically, the review summarizes advances in 3D printing technology for pharmaceutical drug products with specific emphasis on pharmaceutically feasible 3D printing methods. Furthermore, with the aid of a hypothetical case study, the review further explores the role of 3D printing in various pharmacy operations. Finally, it predicts the future of this technology in a newly coined term, “super-compounding” pharmacy. As the pharmacy profession continues to evolve, 3D personalized printed dosage forms can be investigated as a “think outside the box” new approach in pharmacy practice, providing future pharmacists with an avenue to deliver personalized patient care with excellent outcomes.

## 2. Overview of Pharmaceutical 3D Printing

3D printing at a super-compounding pharmacy is the future for this technology, enabling precision and personalized medication, and an opportunity for pharmacists to practice across various settings (independent pharmacies with or without compounding facilities, ambulatory care settings, inpatient and outpatient hospitals, etc.). To leverage this innovation, before focusing on future applications of 3D printing in such specialized mainstream pharmacy practice, it is essential to acknowledge the established research and advancements in 3D printed drug products [7,8,9,10,11,12,13,14]. The intricate knowledge and understanding of such developed 3D printed drug products will enable future pharmacists to bridge the gap between benchside researched 3D printed drug products and actual bedside printing and clinical use.

While many 3D printed pharmaceutical products are still under investigation, some have already reached the market or are undergoing clinical trials. Pharmaceutical scientists worldwide have reported multiple 3D printing techniques for printing drug products. Table 1 lists all such relevant and essential methods along with their key features and related cautions. A significant number of studies in the literature (Table 1) corroborate that 3D printing can be used as a replacement method for developing conventional dosage forms, polypills, etc. Furthermore, by meticulously designing the shape/geometry, structure, density, polymer type, and the ideal 3D printing method, it can also be used as a tool to develop products with a modulated drug release rate, leading to a multitude of immediate, controlled, and sustained release formulations.

Furthermore, although liquid dosage forms can be used for precision dosing by titrating the administered volume, 3D printing is anticipated to offer superior accuracy and patient compliance. This is because liquid dosing may lead to measurement errors due to reliance on user technique, whereas 3D printing enables the production of solid unit dosage forms with high precision and personalized dosing. Additionally, 3D printed drug products can achieve controlled drug release profiles and can provide effective taste masking, which can be challenging in the case of liquid formulations. Moreover, multiple drugs can be combined into a single 3D printed polypill while reducing drug–drug interactions through compartmentalization. Finally, 3D printed dosage forms are more compact than liquid dosage forms and can be customized in shape, size, and flavor to enhance palatability and patient compliance.

Various 3D printing techniques have been explored for pharmaceutical drug products, each offering distinct advantages and limitations depending on the desired target drug product profile (Table 1). Among these, drop-on-demand inkjet printing, extrusion-based printing, fused deposition modelling (FDM), stereolithography (SLA), and selective laser sintering (SLS) represent the most widely investigated approaches (Table 1).

Drop-on-demand inkjet 3D printing (proprietary technologies like ZipDose^®^, TheriForm™, Valvejet etc.) is one of the most established methods in drug product manufacturing. Briefly, this technique uses digitally controlled thermal or piezoelectric print heads for precise droplet deposition to form unique 3D geometry. Key advantages of this technique are the ability to incorporate a wide range of drugs and excipients, high drug loading, diverse geometries, and easy incorporation of multiple drugs within a single dosage form. The method is also readily scalable and has demonstrated clear regulatory feasibility, as evidenced by the USFDA-approved product SPRITAM^®^. These attributes make inkjet printing particularly attractive for personalized medicine and complex dosage form design [13,15,16,19].

Extrusion-based printing techniques, including hot-melt extrusion, direct powder extrusion, and MED, rely on the layer-by-layer extrusion of viscous, semisolid, or molten materials followed by congealing to form stable unit dosage forms. These techniques are optimal for formulations requiring viscoelastic properties and can accommodate complex and multidrug systems. However, they often involve the use of heat and/or solvents, which may limit their applicability for thermolabile drugs. Despite these limitations, extrusion-based approaches remain highly versatile and are commonly used to design customized drug-release profiles [17,18,22].

FDM is a specialized extrusion-based technique in which thermoplastic polymers or excipients are heated to their melting point and deposited layer by layer. FDM is valued for its relatively low cost, good mechanical strength of printed dosage forms, and compatibility with hot-melt extrusion processes. Nevertheless, it requires polymers with suitable thermal properties and drugs that are stable at such processing temperatures. Although the choice of excipients is limited, the use of low melting point polymers has expanded its potential to include certain thermosensitive drugs [21,25,26,35].

SLA is a laser-based technique that employs UV-induced polymerization of photopolymers to form solid structures. This method offers high resolution, smooth surface finish, and the ability to produce dosage forms with unique geometry. The SLA technique does not require high processing temperatures and is therefore considered suitable for sensitive biomolecules, such as proteins and peptides. However, the SLA-based 3D printing is relatively slow, and its wide utilization is restricted by limited availability biocompatible photopolymers [40,46].

SLS utilizes a laser to sinter polymers layer by layer through localized melting and congealing. While it eliminates the need for solvents and can produce robust structures, SLS requires laser-responsive polymers, thereby restricting excipient options. This limitation has slowed its broader adoption in drug product development compared with other 3D printing techniques [44,45].

Among the various methods discussed above and summarized in Table 1, the drop-on-demand inkjet 3D printing method is widely employed due to its pharmaceutical compatibility, ease of operation, and control (the first USFDA-approved drug product SPRITAM^®^ is based on this technique). However, it must be realized that the most optimal 3D printing method must be appropriately selected depending on the drug properties, excipient properties, desired dosage form, etc. For example, thermolabile drugs (proteins/peptides) may not be suitable for 3D printing techniques that utilize high temperature, or in such cases, the temperature should be appropriately controlled to avoid drug degradation. As reported in the literature, SLA or FDM printing is considered a suitable method [34,35]. Furthermore, the use of multiple medications can lead to errors in medication and cause patient non-compliance/non-adherence issues, so combining multiple medications into a unit dosage form (polypill) is a valuable alternative. Interestingly, 3D printing has been widely explored for preparing polypills, as the printing tool can be optimized to achieve different layers and/or compartments of the drug, each with its unique drug release profile. For such applications, drop-on-demand inkjet 3D printing and extrusion-based printing methods are the choices, as reported in the literature.

3D printing has been reported to develop a variety of conventional dosage forms, including tablets of different shapes/layers, capsules, hydrogels, oral films, filaments, extrudates, and scaffolds, etc. A significant number of studies and a wide range of literature, listed in Table 1, corroborate that 3D printing can be definitively used as a replacement method for developing conventional unit dosage forms. Besides conventional drug products, 3D printing can also be used to design and print innovative dosage forms that, in turn, allow the interpretation and future scope of this technology for personalized tailoring. For a better understanding, a few interesting studies are discussed in the following sections.

### 2.1. 3D Printing to Modulate Pharmaceutical Medicine Performance and Drug Stability

3D printing can be used as a tool to modulate the drug release rate, leading to a multitude of immediate, controlled, and sustained-release formulations. For example, Racaniello et al. recently reported 3D printed tunable release orodispersible films of lidocaine for pediatric use prepared using a solvent-free single-step direct powder extrusion (DPE) method. Specifically, the immediate-release films consisted of a Copovidone/polyethylene oxide (VA64/PEO) polymeric backbone, while the controlled-release films consisted of a chitosan-modified polyethylene oxide/hydroxypropyl methylcellulose (PEO/HPMC) polymeric backbone. The studies revealed tunable drug release with superior mucoadhesive properties, making them suitable for pediatric pain management with a patient-centric approach. Furthermore, the tunability offered an avenue for catering to patient-specific needs [20]. In another investigation, 3D-printed alginate-polydopamine hydrogel scaffolds with higher cell adhesion capability were developed for breast cancer treatment and tissue regeneration (post-surgery). The scaffold exhibited high flexibility and a similar modulus to that of the breast tissue, facilitating normal epithelial tissue regeneration. Furthermore, the scaffolds exhibited a photothermal effect both in vitro and in vivo, and were confirmed to have significant efficacy [47]. Shi et al. developed a pH-responsive smart scaffold wherein anticancer drugs, 5-fluorouracil and doxorubicin hydrochloride, were incorporated into polymeric layers of poly(lactic-co-glycolic acid), gelatin, and chitosan. The scaffold exhibited a sponge-like structure that not only inhibited tumor growth and recurrence but also demonstrated good biocompatibility and reduced drug toxicity. Hence, 3D printing can be used as a tool to design and manufacture complex innovative drug products [48]. Additionally, 3D printed hydrogels and scaffolds for the sustained release of dexamethasone, growth factors for wound healing, and imidazo-pyrazoles for melanoma treatment have also been reported [49,50,51]. Interestingly, a study reported a novel 3D. This indicates a wide spectrum of applications for 3D printed scaffolds and, most importantly, the capability of tuning the product design to offer optimum drug release in the context of the patient, disease state, and site of administration.

In another approach, the geometry of a 3D printed tablet has been shown to affect the drug release. For this, paracetamol tablets with different geometries from a single paste-based formulation were formulated using extrusion-based 3D printing. The tablets exhibited tunable drug release profiles even with high drug loading, and the release rate could be changed from immediate to sustained by manipulating tablet geometry and surface area. For example, the mesh geometry 3D printed tablets achieved immediate release of more than 70% of the paracetamol, while the ring and the solid tablets achieved sustained drug release of only 25% and 12% in the same period, respectively. Precisely, it was observed that the drug release increased with an increase in the surface area/volume ratio and the surface area of the tablets [22].

Not only have innovative drug products with modified shapes and release patterns been developed, but 3D printing has also been utilized for many thermolabile drugs [34]. For instance, the thermo-sensitive drug pantoprazole sodium was printed into an immediate release tablet using the fused deposition modeling method at lower temperatures (below 100 °C). For this, five polymer excipients allowing 3D printing at lower temperatures were meticulously selected and tested. In addition, printing design and the process temperatures were also optimized to achieve a stable immediate-release tablet. Among multiple batches tried at 10% drug loading, tablets with PVP K12 demonstrated the fastest in vitro dissolution (dissolution time 10 min), followed by PEG 6000 tablets [39]. Hence, 3D printing offers flexibility and promise in designing a unit dosage form that can be used to stabilize drug incorporation, tune the drug release, and, in turn, enhance its bioavailability and performance.

### 2.2. 3D Printing to Develop Multidrug Medicines (Polypill)

The use of multiple medications can lead to errors in medication and cause patient compliance issues, so combining multiple medications into a unit dosage form (polypill) is a useful alternative [52]. Interestingly, polypills comprising multiple drugs have already been investigated using 3D printing, and FDM technology has been widely explored for such products [52,53,54]. For example, Windolf et al. reported an innovative 3D printed two-component floating filament for Parkinson’s disease management. The first component consisted of an immediate-release polyvinyl alcohol filament for the immediate release of pramipexole, and the second component consisted of an ethylene–vinyl acetate copolymer filament for the sustained release of the drug combination levodopa and benserazide. Importantly, the floating design allowed retention of the filament in the upper gastrointestinal tract, enabling better absorption of levodopa [55]. In another study, Siyawamwaya et al. developed an anti-HIV three-drug (efavirenz, tenofovir disoproxil fumarate, and emtricitabine) fixed-dose combination 24-layered rectangular prism-shaped tablet for controlled release treatment. In an in vivo investigation, the developed tablet confirmed the controlled drug release (delayed T_max_ for all three drugs) compared to the marketed product Atripla^®^ [56]. In another study, a polypill comprising five drugs was developed with three-dimensional extrusion printing. The unique feature of this polypill was a two-compartment design wherein the first compartment comprised two drugs, aspirin and hydrochlorothiazide, for immediate drug release, while the second compartment comprised three drugs, pravastatin, atenolol, and ramipril, for sustained drug release. Hence, 3D printing can not only allow the incorporation of multiple drugs into one tablet but can also allow the simultaneous modulation of drug release [28].

In our vision, the successes of pharmaceutical research in the development of 3D printed polypills can be extrapolated to the dispensing pharmacy facilities. Similar principles can be used at the dispensing facility by pharmacists to print a personalized polypill that will enhance patient compliance (one polypill instead of multiple tablets) and thereby increase patient adherence to the treatment.

Ultimately, the literature not only demonstrates the great potential of 3D printing in developing demand-based medicines but also in advancing the field of personalized medication with its versatility in the production of various dosage forms of medication.

Another key factor to understand is the 3D printing operation process. Specifically, though each technique may differ in its principle of operation, the standard step-by-step process is similar and is summarized in Table 2. As described in Table 2, the key to quality 3D printed pharmaceutical products is identifying a suitable printing method and using computer-aided design (CAD) software to determine the 3D structure of the unit dosage. Hence, a pharmacist must be trained on both aspects to ensure the successful implementation of 3D printers in the pharmacy setting. Therefore, our firm belief is that the amalgamation of pharmacy knowledge with the 3D printing technology skills will result in the next generation of super-compounding pharmacists.

## 3. 3D Printed Pharmaceutical Drug Products: Pharmacist Practitioner Perspective

**Figure 1:**
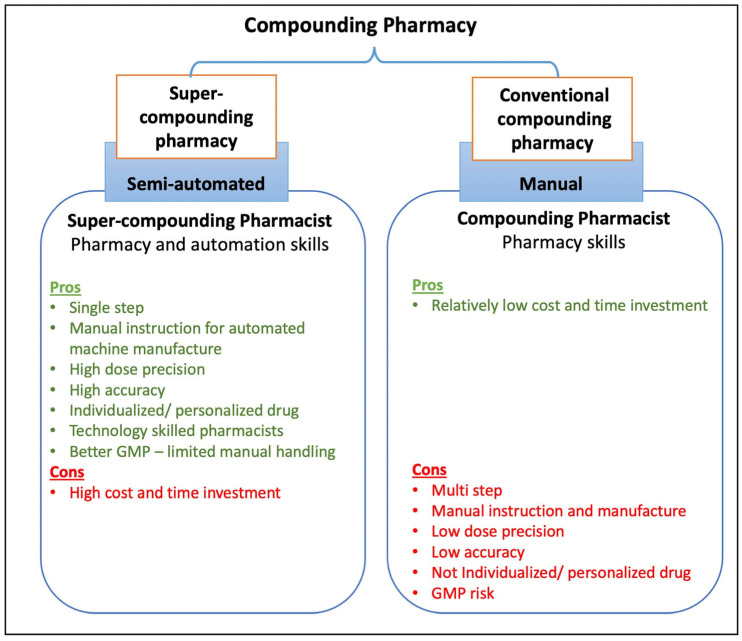
Comparison of 3D printing super-compounding pharmacy with conventional compounding pharmacy.

In our vision, the preclinical, clinical, and industrial success of 3D printed drug products can be extrapolated to the pharmacies to print on-site personalized medication for patients. Such super-compounding pharmacies will comprise trained pharmacists and an on-site 3D printer equipment facility that will enable on-site manufacture (printing) of single drug/multidrug formulations based on the patient’s prescription. This can be visualized with the help of Figure 1, which compares the proposed super-compounding pharmacy against the conventional compounding pharmacy. Inclusion of 3D printing technology in the pharmacy will not only ensure individualized prescription manufacture, but it will also ensure higher accuracy and precision, along with improved current good manufacturing practices (cGMPs) due to flexible automation. Additionally, this approach will reinvent the pharmacy profession, enabling pharmacists to compound technologically advanced drug products.

**Figure 2:**
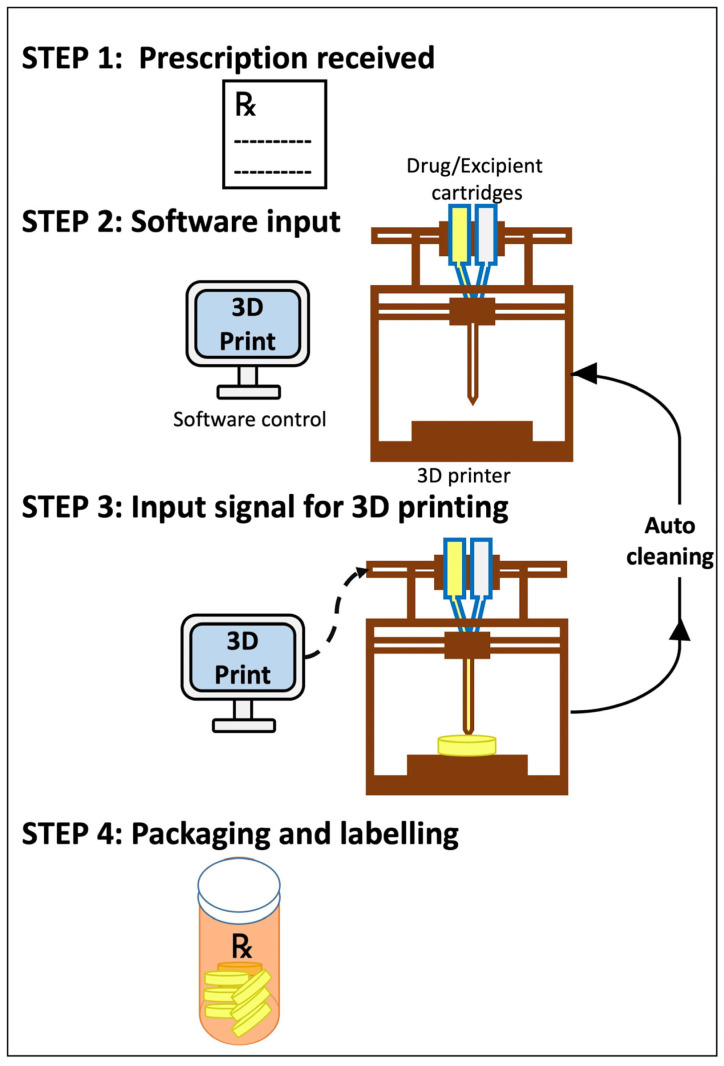
Schematic representation of the step-by-step approach of 3D printing at the super-compounding pharmacy.

For better understanding, the step-by-step approach of the 3D printing super-compounding pharmacy has been depicted in Figure 2. As a hypothetical example, a patient brings a prescription for atorvastatin (a cholesterol management treatment) to the super-compounding pharmacy, and the prescribed dose is 20 mg per tablet. However, following an assessment of his medical history, age, sex, and physiological parameters like weight and BMI (Step 1), it is determined that the actual dose needed for efficient management of his cholesterol condition is 14.4 mg only. This triggers the super-compounding pharmacist response to manufacture the prescribed tablet using a 3D printer for precision dosing of 14.4 mg. The trained pharmacist enters the patient information, drug, dose, and excipient details into the 3D printer (Step 2). The 3D printing device can be visualized as a regular printer wherein the ink cartridges are replaced with the drug/excipient-filled cartridges required for printing of pharmaceutical pills/tablets, etc. The printer follows the commands and prints individual tablets using the atorvastatin cartridge and the excipient cartridge (Step 3), ensuring dosage accuracy. The printed tablets are then packed, labelled, and dispensed to patients by the super-compounding pharmacist, along with special counseling guidelines meant only for the patient for whom it was manufactured. This step enhances the pharmacist’s image as the medication therapy expert (Step 4). The 3D printer undergoes an automated cleaning protocol and is ready to print the next prescription. Using this technology, the super-compounding pharmacist has the flexibility to adjust the dose or to add multiple drugs in the same pill/tablet (polypill) for patient compliance. Below is a relevant case study that discusses the future application of 3D printing in patient care management for a better understanding.

## 4. Polypill Case Study Using 3D Printing Super-Compounding Pharmacy and Medication Therapy Management

**Figure 3:**
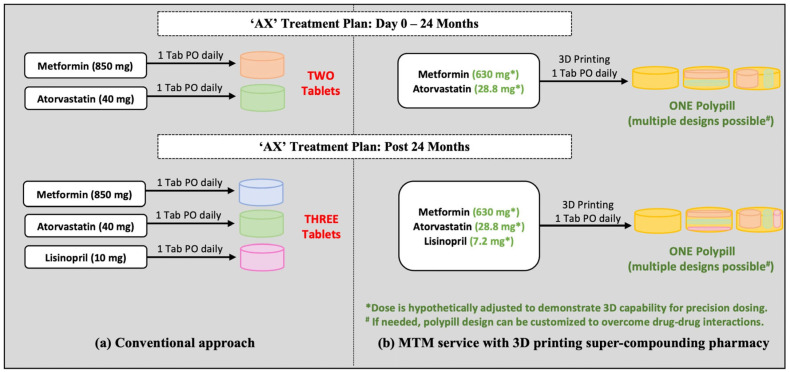
Multiple chronic diseases management plan for patient AX by (**a**) conventional approach, (**b**) MTM service using 3D printing super-compounding pharmacy approach.

This is a ‘hypothetical case study’ wherein we assume that 3D printing super-compounding pharmacies are functional. The case study focuses on patient AX who is a 63-year-old male. On Day 0, AX was diagnosed with diabetes mellitus and hyperlipidemia. At that point, AX was prescribed two types of conventional medications, IR metformin (850 mg—1 Tab PO) and atorvastatin (40 mg—1 Tab PO), to manage his conditions, diabetes mellitus and hyperlipidemia (Figure 3a). AX started the treatment but was not comfortable with multiple medications per day (Figure 3a), and as a result, he could not adhere to the treatment. AX approached the physician with his concern regarding the lack of medication adherence due to increased pill burden (Figure 3) and was also worried about potential side effects.

AX’s suboptimal adherence to his medications triggered his health plan to refer him to a Medication Therapy Management (MTM) pharmacist to address this issue. After discussing with AX and to increase AX’s medication adherence, the MTM pharmacist decided the best option was to compound multiple medications (metformin and atorvastatin) for his chronic disease states (diabetes mellitus, hyperlipidemia) into a single tablet utilizing 3D printing to reduce the pill burden. Additionally, from the precision medication perspective, assessment of his medical history, age, sex, and physiological parameters like weight, BMI, etc., was conducted, and it was determined that the actual dose needed for efficient management of his conditions was lower than the commercially available individual medications. As a first step, the dose of both the drugs was adjusted considering precision dosing in accordance with patient parameters that resulted in an effective dose requirement of metformin (630 mg—1 Tab PO) and atorvastatin (28.8 mg—1 Tab PO). Using these reduced doses, a single tablet comprising both the drugs metformin and atorvastatin was printed using 3D printing technology (Figure 3b).

This was possible because AX’s physician and the pharmacist already had a collaborative practice agreement (CPA) implemented. With this approach, instead of two types of tablets to manage AX’s health, the MTM pharmacist decided to manage AX’s condition with just one polypill at a reduced dose (precision medicine). AX has been under the care of the MTM pharmacist for the past two years, and AX’s adherence has improved with the reduced number of pills. This has a positive impact on his health outcomes and the health plan’s performance in terms of quality measures (AX’s treatment plan is summarized in Figure 3b).

After 24 months, AX was newly diagnosed with hypertension. AX was then referred to the MTM pharmacist by the physician for further management of his hypertension. AX visited the pharmacist to follow up with his current chronic illnesses and further manage his newly diagnosed hypertension. At this visit, the MTM pharmacist confirmed that AX’s diabetes mellitus and hyperlipidemia were well-maintained and at goal. After discussing AX’s recently diagnosed hypertension, the pharmacist decided to initiate AX on lisinopril (10 mg—1 Tab PO). As part of the MTM program, AX was also educated on lifestyle modifications such as diet and exercise to improve his hyperlipidemia and hypertension. During the discussion, AX again expressed his concern that his newly diagnosed hypertension would increase his pill burden and was worried about potential treatment adherence. To address the issue of increased pill load, the MTM pharmacist decided to modify the treatment plan by compounding multiple medications (metformin, atorvastatin, lisinopril) for his chronic disease states (diabetes mellitus, hyperlipidemia, hypertension) into a single tablet utilizing 3D printing to reduce the pill burden. Additionally, from the precision medication perspective, the dose adjustment was carried out wherein the effective doses of metformin (630 mg—1 Tab PO) and atorvastatin (28.8 mg—1 Tab PO) were retained from the previous polypill as the patient’s pre-existing conditions were well maintained, and the dose of lisinopril was adjusted to 7.2 mg—1 Tab PO. Using these reduced doses, a single tablet comprising three drugs, metformin, atorvastatin, and lisinopril, was printed using 3D printing technology (Figure 3b).

With this approach, instead of three types of tablets to manage AX health, the MTM pharmacist decided to manage AXs condition with just one polypill at a reduced dose (precision medicine). The final plan to manage AX’s health during the MTM pharmacist meet is summarized in Figure 3b.

### Case Study Discussion

AX’s hypothetical case illustrates the utility of 3D printing of medications and the application of precision medicine. Instead of taking multiple pills, with the help of an MTM pharmacist, AX could manage his conditions with one 3D printed pill chronically (at a reduced drug dose—Figure 3). Interestingly, even after diagnosis of a new chronic illness over the period, AX is still taking only one 3D printed pill chronically with the addition of a new medication (at reduced drug dose—Figure 3).

This innovative approach is another solution that pharmacists could use to address adherence issues as part of a Medicare Part D plan’s MTM program. A Medicare Part D sponsor under the Centers for Medicare and Medicaid Services (CMS) is required to establish an MTM program. The goals of MTM programs are to improve medication use and reduce adverse events. Given the goals of the program to improve therapeutic outcomes while minimizing adverse reactions in combination with the opportunities for pharmacists to directly interact with patients, 3D printing of medications to dose precision aligns with the goals of MTM.

Furthermore, CMS employs a star rating method to indicate the quality of the Medicare plans. This rating is determined by the Medicare plan’s performance in various quality measures. These measures include patient adherence to specific classes of medications (i.e., diabetes medications, statins, RAS antagonists). 3D printing may alleviate the pill burden and improve adherence by safely combining medications. It would provide an additional tool to improve patient outcomes further and improve plan performance.

In a practical scenario, a patient would have their laboratory blood work and urinalysis (i.e., comprehensive metabolic panel, comprehensive blood panel, renal function, lipid levels, thyroid function) completed just prior to their visit with an MTM pharmacist. Collaborative practice agreements (CPAs) between a licensed healthcare provider and pharmacist allow pharmacists to initiate, adjust, monitor, and continue drug regimens (Collaborative Practice Agreements and Pharmacists’ Patient Care Services). The pharmacist reviews the patient’s laboratory results to help guide any necessary adjustments to the patient’s drug regimen to achieve the patient’s goals. With any changes in medications or dosages, an MTM pharmacist trained in 3D printing can proceed to combine medications to reflect these changes.

However, there are some potential challenges. First, CMS would have to approve the 3D printing of medications as part of the program. Second, depending on state laws, the MTM pharmacist may need to establish a CPA with healthcare providers. (Pharmacists in many states currently practice under CPA to provide care for patients.) Lastly, MTM pharmacists would need to be trained in 3D printing of medications. In addition, incorporating multiple drugs into a single 3D printed polypill may require careful consideration to overcome known or potential drug–drug incompatibility and stability issues. In this regard, pharmacist knowledge and 3D printing technology can offer a significant advantage. Specifically, we recommend the use of individual drug and excipient cartridges to prevent drug contact prior to printing. Furthermore, a trained super-compounding pharmacist can meticulously design a polypill to allow spatial separation of drugs within the 3D printed polypill to mitigate such interactions and stability issues. This concept is illustrated in Figure 3b. and discussed in detail in Section 5.1. Overall, these challenges are amenable in a way that the pharmacist can be trained to be an expert in the field of 3D printing, and with the help of legislative reforms, a 3D-printing super-compounding pharmacist (MTM or otherwise) can be the future of this field.

## 5. Challenges of 3D Printing

### 5.1. Regulatory Challenges

As discussed in previous sections, 3D printing of drug products will enable precise dosing and personalized formulations, moving away from the traditional ‘one-size-fits-all’ model. Allowing super-compounding pharmacies to print the drug products would definitely benefit patients, empower pharmacists, revitalize the profession, and strengthen their role in delivering personalized patient care. However, it is vital to consider both the printed drug product quality and safety, and the burden of accountability on the frontline pharmacy practitioners.

This is important because the proposed 3D printing super-compounding approach contrasts with the long-standing reliance on licensed and regulated industrially manufactured drug products, which in turn reduces the accountability pressures on frontline practitioners. Introducing drug product manufacturing into super-compounding pharmacies within the proposed scope may increase complexity, variability, and professional liability if appropriate safeguards are not in place. The success of this vision depends on the development and implementation of strict regulatory guidelines along with additional training to prepare pharmacy practitioners for advanced super-compounding roles. Overall, this approach has the potential to benefit patients through improved efficacy, reduced dose-related side effects, and better compliance and adherence. Therefore, it should be embraced with a positive outlook alongside careful regulatory oversight, and standardization to ensure quality and safety. This can be achieved by implementing appropriate GLP handling practices. Currently, USP General Chapter <795> specifies standards for non-sterile compounding pharmacies to ensure the quality of the products and safety of the patients. Super-compounding pharmacies will similarly be required to follow these standards and any policies set forth by the State Board of Pharmacy for non-sterile compounding pharmacies. Moreover, for quality control and assurance, sufficient testing and generation of master formulation for 3D printable drugs should take place. Regulatory bodies worldwide are also working to streamline quality, safety, and efficacy assurance of 3D printed pharmaceuticals. In this context, 3D printing of pharmaceutical devices has been widely accepted by the regulatory bodies [57,58,59], while the area of 3D printed dosage forms still remains relatively new, awaiting specific guidelines (only one product has been approved so far by the USFDA). Additionally, a clear distinction between manufacturing and compounding must be drawn. Currently, the USFDA states that the compounded products cannot essentially be a copy of a commercial product (same API, route of administration, same/easily substitutable dosage, etc.) [60]. Such regulations will be necessary to prevent the super-compounding pharmacies from producing unapproved generic compounds.

Importantly, cartridge-based pharmaceutical 3D printing holds considerable promise for personalized medicine, but it may also present some scientific, regulatory, and industrial challenges. Key concerns include controlling access to APIs, ensuring the quality and safety of drugs and excipients, and balancing the impact on the pharmaceutical industry while promoting innovation. In our vision, a practical and sustainable solution is for the pharmaceutical industry manufacturers to supply licensed, quality-controlled drug and excipient cartridges designed specifically for 3D printing. Allowing pharmaceutical industry manufacturers to prepare and supply such cartridges will not only keep the pharmaceutical industry thriving but will also ensure quality and safety, as the cartridges will be produced in compliance with regulatory specifications and cGMPs. Such cartridge development will need meticulous standardization and control to ensure API stability, long-term compatibility, and overall product quality. This is possible by implementing robust quality control, validated expiry dating, defined storage conditions, and potentially new USP monographs or FDA guidance specific to 3D printing cartridges. Finally, once such cartridges are received at the 3D super-compounding pharmacy, the trained pharmacist can design, print, and dispense the 3D printed personalized drug products to patients.

Cross-contamination can be another challenge. To prevent issues of contamination and minimize drug–drug or drug–excipient interactions before 3D printing, we envision that pharmaceutical industry manufacturers should pack each drug and excipient individually in 3D printer-compatible cartridges, analogous to individual color cartridges in a conventional printer. Additionally, this will also allow a seamless 3D printing operation at the compounding facility, as the cartridges can be attached to the printer as needed. This setup will offer three advantages: 1. retain quality and purity while reducing cross-contamination; 2. simplify 3D printing operations with easy cartridge management; 3. enhance regulatory control by ensuring quality standards.

Moreover, multi-drug polypills (as proposed and discussed in the hypothetical case study and Figure 3b) with known drug–drug incompatibility/interaction will also need special attention. In this context, the combination of individual drug and excipient cartridges and 3D printing may offer a significant advantage. Storing each drug separately in a cartridge ensures that there is no direct contact between interacting drugs prior to printing. Furthermore, for such drug combinations, unique 3D printing software and design capabilities can be advantageous, as they can be used to design and print specialized 3D unit dosage forms that allow spatial separation of interacting drugs within a layered or compartmentalized unit dosage form. For the polypills comprising multiple drugs with unrecognized or unreported drug–drug interactions, a similar approach can be implemented as a precautionary measure to prevent any possible drug–drug incompatibility or stability issues inherent to combining multiple drugs.

Furthermore, strict regulatory requirements and technological considerations should be placed when using 3D printing for potent and/or high-risk drugs (habit-forming drugs posing drug abuse issues, anticancer medications, antibacterial, antiviral agents, etc.). For example, in 2015, the USFDA released a guidance for industry, highly recommending the production of opioid medications with abuse-deterrent properties [61]. Hence, 3D printing technology to be used with the habit-forming medications would require a more specialized 3D printer that can incorporate abuse-deterrent properties. Using a 3D printer for anticancer medications and antimicrobial agents can also be challenging due to the potential cross-contamination. Trace amounts of anticancer medications left on the device can lead to unwanted exposure and adverse reactions in other patients. With antibiotics and antivirals, cross-contamination may develop antimicrobial resistance. Hence, the use of 3D printing should focus on the drugs for long-term illnesses or conditions that may increase the pill burden. Moreover, pharmacy personnel must ensure thorough device operation and cleaning. Strict regulations with compounding multiple active ingredients set by the USFDA also need to be addressed to produce polypills with a 3D printer. Currently, the USFDA does not permit compounding of drug products with multiple active ingredients with strengths available in commercialized products due to the potential drug interactions and formulation complications [60]. Installation of the program, which can detect the harmful interactions, will be necessary, along with specialized pharmacist training.

### 5.2. Cost and Infrastructure Considerations

We must consider the overall cost and space for super-compounding pharmacies. Multiple GLP pharma-grade 3D printers are necessary for various dosages, which are more expensive than non-pharma options. Adequate spacing is essential to prevent 3D printer overheating and cross-contamination. Furthermore, as discussed in the earlier section, we envision that the supplier companies would pack drugs and excipients in individual cartridges, which may require a larger space at the compounding pharmacy facility for storage. Furthermore, machine-aided retrieval of necessary chemical cartridges may be beneficial so that different 3D printing devices can use those cartridges as needed.

Hence, we anticipate an increase in early-stage investment in the set-up of such super-compounding pharmacies. However, such establishments will have an early break-even point and profitability due to the forecasted increase in demand and supply as personalized medication will become mainstream in the next decade. Furthermore, the cost of 3D printed medication to the patient should also be considered. In the early stages of 3D printing super-compounding pharmacies, these medications may become more expensive than conventional products (high initial investments and relatively low demand). In such scenarios, the rationale for selecting 3D printed medications must be evaluated on a case-by-case basis through patient-specific cost-benefit analysis (e.g., high-risk drugs, 3D printed polypills to address treatment non-adherence, pill burden, overall cost of multiple pills, etc.). However, once established, the cost of 3D printed medications will decline over the years with increased acceptance and higher demand.

Another aspect to consider with a 3D printing facility is the scenario wherein the patients fail to pick up the prescription. For instance, according to two-month research performed by Shrank et al., about 3.3% of 10,349,139 filled prescriptions (~341,521 prescriptions) were never picked up by the patients [62]. In traditional pharmacies, prescription medications that are not picked up by the patients can be used to fill other patients’ prescriptions (one dose fits all). However, with the personalized dosages and polypills, these medications will most likely be discarded, increasing the management cost. Possibly, implementing a system where the prescription is filled only at the patient’s request, with a scheduled pick-up time, may be needed. This may also increase the patient’s medication adherence and therapeutic success as a result.

## 6. Discussion and the Future of 3D Printing

3D printers will be the next breakthrough in pharmacy practice, emerging as a super-compounding pharmacy practice with more career opportunities for future pharmacists. For instance, a 3D printing-focused pharmaceutical company, FabRX, has already completed its first-of-its-kind clinical study using 3D printlet technology and plans to establish an in situ personalized medicine 3D printing facility in hospitals by 2027 [5].

On the compounding front, we envision that early on, 3D printing super-compounding pharmacies can become mainstream in Ambulatory Care and MTM practice site settings for the management of common and chronic diseases like diabetes, hypertension, hyperlipidemia, etc. This transition will be safer, faster, and smoother, considering high demand, chronic management requiring patient treatment adherence measures, and relative safety of medications. Once the scope of 3D printing is established, super-compounding pharmacies can be extrapolated to other disease management approaches.

In addition to pharmacist training, other challenges, including legislative reform in terms of specific guidelines comprising compounding laws and regulations for 3D printing, must be implemented under the compounding umbrella for the future translation of this technology in pharmacy practice.

## 7. Conclusions

Overall, the 3D printing technology as a personalized medication approach has significant potential and is achievable by a multipronged approach that includes a super-compounding pharmacy setup, pharmacist training, support from regulatory authorities, and the pharmaceutical industry. Most importantly, we strongly believe that this will reinvent the pharmacy profession by not only creating more job opportunities for pharmacists but also allowing personalized care for patients, leading to better health outcomes.

## Figures and Tables

**Table 1:** Pharmaceutically relevant 3D printing techniques and current preclinical and clinical applications [3,4,12,13,14,15,16,17,18,19,20,21,22,23,24,25,26,27,28,29,30,31,32,33,34,35,36,37,38,39,40,41,42,43,44,45].

3D Printing Technique	Equipment/ Technology	Key Features	Dosage Form	Drug
**Drop-On-Demand Inkjet 3D Printing** (Substrate Dependent: Drop-On-Solid/ Drop-On-Powder)	Zipdose Technology (drop-on powder) TheriForm^TM^ Valvejet	Allows combination of drugs and excipients Digital control enabling small droplets sprayed on a substrate Printer head (thermal/ piezoelectric) Wide choice of excipients Microscale dosage form High drug loading capacity Varied geometry possible Fast disintegration Possible multiple drugs formulation Easy scalability Marketed product available	SPRITAM^®^ tablet	Levetiracetam
			Oral film	Salbutamol sulphate
			Solid dosage forms	Prednisolone, paracetamol, indomethacin
			2-drug polypill- controlled release	Propranolol hydrochloride, riboflavin sodium phosphate
**Extrusion-Based** **Printing**	Global extrusion Hot-melt extrusion Direct powder extrusion Melt extrusion deposition (MED)	Extrudes viscous/semisolid/solid material layer by layer Suitable viscoelastic properties are desired Solvents and/or heat are used Suitable for complex drug formulations Suitable for multiple drug formulations	Encapsulated system	Dexamethasone- 21-phosphate disodium salt
			Geometry-optimized modified release tablet	Paracetamol
			3pdrug polypill	Nifedipine, glipizide, captopril
			5-drug polypill	Hydrochlorothiazide, aspirin, pravastatin, atenolol, ramipril
			Filaments	Theophylline
			Oro- dispersible Film	Lidocaine
			T20G microstructure Gastro- retentive	Non-vitamin K antagonist oral anticoagulant
			T21 tablet, colon targeted	Tofacitinib
**Fused Deposition Modelling (FDM) Printing**	Ultimaker 2 Protos v3	Polymer/excipient heated to its melting point followed by layer-on layer extrusion. Polymer/excipient should have suitable thermal properties (thermoplastic polymers) Drugs should be thermostable at processing temperatures Used for thermosensitive drugs (polymers with low processing temperatures as excipients) Relatively low cost High mechanical strength Limited choice of excipient polymers Can be combined with hot-melt extrusion	Tablet	Felodipine, Glipizide, 5-Amino Salicylic Acid, Captopril, Theophylline, Prednisolone, Pantoprazole, Enalapril Maleate
			Orodispersible tablet	Fluconazole
			Modified release tablet	Budesonide
			Shell-core delayed release tablet	Theophylline, Budesonide, Diclofenac Sodium
			Extended release tablet	Acetaminophen
			Bilayer tablet	Metformin and glimepiride
			Intragastric floating tablets	Domperidone, theophylline
			Oral film	Aripiprazole
			Scaffold	Ibuprofen
			Extrudate	Haloperidol
**Stereolithography (SLA) Printing**	Xometry™ 3D stereolithography	Laser-based technique Suitable for liquid photopolymers with rapid UV-induced polymerization Suitable for proteins and peptides Possible smooth surface and micro size Limited choice of excipient polymers Relatively slow process	Tablet	Paracetamol
			Tablet	4-Aminosalicylic acid and paracetamol
			Hydrogel	Ibuprofen
**Selective Laser** **Sintering (SLS)** **Printing**	Xometry™ SLS 3D printing	Sintering (melting and congealing) is achieved by means of laser Layer-by-layer sintering requires laser-responsive polymer Limited choice of excipient polymers.	Tablet	Paracetamol
			Device	Progesterone

**Table 2:** Step-by-step protocol of standard 3D printing process.

Step	Description
1	Select an appropriate 3D printer method and equipment (Refer to Table 1).
2	Obtain 3D printer compatible cartridges that are individually loaded with drugs and excipients.
3	Assemble the cartridges in a 3D printer that is connected to computer-aided design (CAD) software.
4	Use CAD software to determine the 3D structure of the unit dosage from like geometry, color, and composition, etc.
5	Transform the CAD into a printer-friendly file format (.STL/.OBJ).
6	3D-print the unit dosage form followed by packaging and labelling (refer to Figure 2).
7	Counsel the patient on proper use of the individualized dosage form the pharmacist has created.

## Data Availability

No new data were created or analyzed in this study.

## Abbreviations

The following abbreviations are used in this manuscript:

3D	Three-dimensional
USFDA	U.S. Food and Drug Administration
ISO	International Standard Organization
FDM	Fused deposition modelling
SLA	Stereolithography
SLS	Selective laser sintering
VA64	Copovidone
PEO	Polyethylene oxide
HPMC	Hydroxypropyl methylcellulose
PVP	Polyvinyl pyrrolidone
PEG	Polyethylene glycol
CAD	Computer-aided design
MTM	Medication therapy management
PO	Per oral
CMS	Centers for Medicare and Medicaid services
CPA	Collaborative practice agreements
GLP	Good laboratory practices
IND	Investigational new drug
MED	Melt extrusion deposition
